# Heat shock protein 27 downstream of P38-PI3K/Akt signaling antagonizes melatonin-induced apoptosis of SGC-7901 gastric cancer cells

**DOI:** 10.1186/s12935-016-0283-8

**Published:** 2016-02-12

**Authors:** Wenjie Deng, Yujie Zhang, Luo Gu, Jie Cui, Biao Duan, Yueyuan Wang, Jun Du

**Affiliations:** Cancer Center, Nanjing Medical University, 140 Hanzhong Road, Nanjing, Jiangsu 210029 China; Department of Physiology, Nanjing Medical University, 140 Hanzhong Road, Nanjing, Jiangsu 210029 China; Department of Biochemistry and Molecular Biology, Nanjing Medical University, 140 Hanzhong Road, Nanjing, Jiangsu 210029 China

**Keywords:** Melatonin, P38, PI3K/Akt, HSP27, Gastric cancer

## Abstract

**Background:**

Despite the fact that melatonin treatment shows some promise in gastric cancer, the molecular mechanisms of gastric cancer cells in response to melatonin remains to be determined.

**Methods:**

The SGC-7901 gastric cancer cells were treated with different concentrations of melatonin for 24 and 48 h. Cell viability was determined by MTT assay, Hoechst 33258 staining and FACS analysis were used to detect apoptotic cells. The contents and activation of apoptosis-related proteins HSP27, Akt and P38 were evaluated by immunoblotting analysis. Then we treated SGC-7901 cells with HSP27-specific siRNA, PI3K inhibitor LY294002 or P38 inhibitor SB203580 to investigate the role of HSP27, Akt and P38 in the anti-apoptotic response of SGC-7901 cells to melatonin.

**Results:**

Melatonin suppressed cell viability and stimulated apoptosis of gastric cancer SGC-7901 cells dose-dependently. Mechanistically, the observed apoptosis was accompanied by the melatonin-induced phosphorylation of HSP27. HSP27-specific siRNA transfection effectively reduced HSP27 phosphorylation and augmented melatonin-induced apoptosis, indicating that HSP27 is resistant to melatonin-induced apoptosis. Moreover, melatonin increased PI3K/Akt activation, LY294002 abrogated HSP27 activation and promoted cell apoptosis induced by melatonin. Furthermore, melatonin increased P38 activity, and P38 inhibitor SB203580 inhibited melatonin-induced PI3K/Akt, HSP27 activation and accelerated cell apoptosis.

**Conclusion:**

In contrast to the well-established anti-cancer properties of melatonin, our study revealed clearly a distinguishable anti-apoptotic pathway induced by melatonin, that is, HSP27 plays a crucial role in apoptotic resistance in melatonin-treated gastric cancer cells, and its activation is most likely via the activation of P38/PI3K/Akt signaling.

## Background

Gastric cancer has become the fourth most common malignancy and the second in mortality of total cancer worldwide [1]. It is estimated that in 2012 there were 951,600 new cases and 723,100 deaths from gastric cancer in the world [2]. Despite the development of therapeutic intervention for gastric cancer in clinical trials, drug resistance is still the major reason why failure occurs in gastric cancer treatment. As reviewed recently, melatonin, an indolamine derived from the l-tryptophan, has been shown to exert important protective effects in the gastrointestinal tract [3]. Although further study in additional settings and populations is required, melatonin is of great interest among the compounds tested as a potential therapy in gastric cancer.

Melatonin is an endogenous hormone that is required for the daily onset of darkness. Besides its use in the treatment of some sleep disorders, melatonin has been characterized as possessing a pro-apoptotic role in the treatment of cancer, and there seems to be some promise in several types of cancers such as breast, prostate as well as colorectal cancer [4–6]. There is at least 400 times more melatonin produced in the gastrointestinal tract than in the pineal gland [7], and melatonin has been demonstrated to suppress gastrointestinal carcinogenesis under some circumstances such as chronic inflammation [8, 9]. The safety of melatonin heralds it as a great prospect for gastrointestinal cancer therapy [3], however, the molecular mechanism related to its effect on gastric cancer remains unelucidated.

HSP27, a member of the small heat shock protein (HSP) family, is believed to have tumorigenic and pro-metastatic functions, characterized by its dynamic phosphorylation leading to heterogeneous oligomerization under different conditions such as oxidative stress, heat shock as well as chemical stress [10]. In fact, epidemiologic surveys have demonstrated that HSP27 is upregulated in many cancers including ovarian cancer, colorectal cancer and gastric cancer, where it has been identified as a tumor prognostic marker [11–13]. HSP27 is also closely correlated with increased resistance to apoptosis in stressed cancer cells. For example, HSP27 accumulation reduces the chemosensitivity induced by vincristine and adriamycin agents in gastric cancer cells [14]. In contrast, a combination of traditional chemotherapeutic agents (cisplatin, gemcitabine) and HSP27 inhibitor (quecertin) exerts a surprising greater chemotherapeutic effect in lung stem like cells [15]. Thus, it is worthwhile to explore whether HSP27 is involved in melatonin-induced apoptosis of gastric cancer cells.

Recent studies from our laboratory showed that HSP27 phosphorylation could be induced by P38 activation and phospho HSP27 acts as a regulator of cell cytoskeleton reorgnization and cell adhesive ability [16]. In addition, HSP27 has been found to direct chaperoning interaction with Akt, and protest adenocarcinoma cells from UV-induced apoptosis [17]. Based on above information, we focused this study on HSP27 regulation of apoptosis induced by melatonin, and in an attempt to gain further mechanistic insights into the molecular pathways leading to melatonin-induced alteration of HSP27 phosphorylation in SGC-7901 gastric cancer cells.

## Methods

### Cell culture

Human gastric cancer cell line SGC-7901 was obtained from the Cell Biology Institute of Chinese Academy of Sciences (Shanghai, China). SGC-7901 cells were cultured in Dulbecco’s modified Eagle’s medium (DMEM, high glucose) (Hyclone, Thermo Scientific, Waltham, MA, USA) supplemented with 10 % (v/v) fetal bovine serum (FBS) (Hyclone) and antibiotics (100 U/mL streptomycin and 100 μg/mL penicillin) (Invitrogen, USA) in a humidified incubator at 37 °C with 5 % CO_2_. Cells were grown on coverslips for fluorescence staining and on plastic dishes for protein extraction.

### Treatment and transfection

Melatonin (Sigma, St Louis, MO, USA) was dissolved in ethanol and cells were treated with melatonin for the indicated times and doses. In experiments to determine the effects of inhibitors, LY294002 (Sigma) and SB203580 (Beyotime, Nantong, China) on cell growth inhibition and apoptosis, cells were treated with these kinase inhibitors for 30 min prior to melatonin treatment.

The sequences of small interfering RNA (siRNA) for HSP27 was 5′-UGAGAGACUGCCGCCAAGUAA-3′, the sequence of control siRNA was 5′-UUCUCCGAACGUGUCACGUTT-3′ (GenePharma Co., Shanghai, China). Cells were transfected with control siRNA or HSP27 siRNA with Lipofectamine 2000, according to the manufacturer’s instruction.

### Immunoblotting analysis

Subconfluent cells were washed with PBS, and lysed with RIPA lysis buffer (150 mmol/L NaCl, 50 mmol/L Tris–HCl (pH 7.4), 1 % Triton X-100, 1 % sodium deoxycholate, 0.1 % SDS) with 1 mM sodium orthovanadate, 1 mM PMSF, and 1 % cocktail of protease inhibitors (Sigma). The lysates were clarified by centrifugation at 12,000*g* for 20 min at 4 °C and separated by SDS-PAGE followed by transfer onto nitrocellulose membranes. The following antibodies were used: rabbit anti-P38 antibody, rabbit anti-P-P38 antibody, rabbit anti-P-Akt antibody, mouse anti-HSP27 antibody and rabbit anti-P-HSP27 antibody (Cell Signaling, Danvers, MA, USA), rabbit anti-Akt antibody (Bioworld, Louis Park, USA), rabbit anti-GAPDH antibody (Santa Cruz, CA, USA). Protein bands were detected by incubating with horseradish peroxidase-conjugated secondary antibodies (Santa Cruz) and visualized with ECL reagent (Millipore, Billerica, MA, USA). Digital images of immunoblots were obtained with a Chemidoc XRS and analyzed using the image analysis program Quantity One (Bio-Rad, Hercules, CA, USA).

### Hoechst staining

Hoechst 33258 dyes (Beyotime) are cell permeable nucleic acid stains, which are useful for the recognition of DNA damage and cell apoptosis by monitoring the emission spectral shifts of the dyes. Cells were stained with Hoechst 33258 (5 μg/mL) in PBS for 30 min at room temperature, and then washed to remove unbound dye. Observation and photography was performed in a fluorescence microscope (Olympus BX 51, Tokyo, Japan).

### Flow cytometry analysis

Cells were trypsinized and resuspended in 1× binding buffer, double-stained with Annexin V-FITC and propidium iodide (Beyotime) at room temperature for 15 min in darkness. Subsequently, the stained cells were analyzed by flow cytometry for apoptotic analysis according to the manufacturer’s protocol.

### Cell viability assay

Cell viability was determined by 3-(4,5-dimethylthiazol-2-yl)-2,5-diphenyltetrazolium bromide (MTT) assay as described previously [18]. In brief, SGC-7901 cells were seeded at a density of 5 × 10^3^ cells per well into 96-well plate and treated with melatonin for the indicated times and doses. After culture, cells were washed, MTT was added and the plate was incubated in the dark for 4 h, followed by measurement at 490 nm using a microplate absorbance reader (Bio-Tek, Elx800, USA). The percent cell viability was calculated as the absorbance of melatonin treated sample/control sample absorbance ×100 %.

### Statistical analysis

Data were analyzed by Image J and statistical analyses were carried out using the SPSS software version 15.0 (SPSS Inc., Chicago, IL, USA). Student’s *t* test was used to analyze differences between two groups. Statistical significance was considered when *P* < 0.05.

## Results

### Melatonin promotes gastric cancer cell apoptosis in vitro

**Fig. 1:**
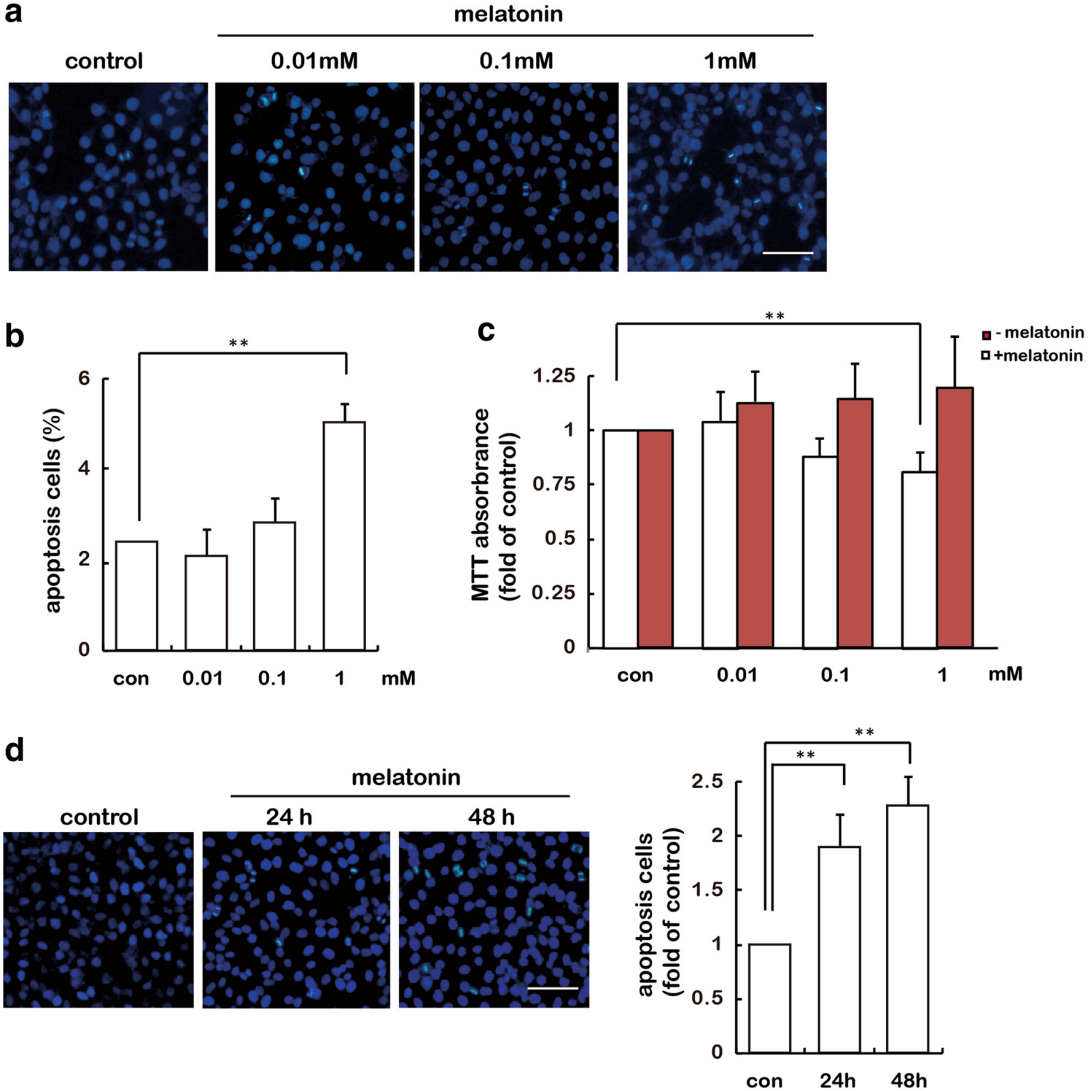
Melatonin promotes apoptosis of SGC-7901 gastric cancer cells. SGC-7901 cells were incubated with melatonin for the indicated doses and periods. **a** The morphological figures of apoptosis were monitored by fluorescence microscopy after staining with Hoechst 33258. Images are representative of at least three independent determinations. *Scale bar* 50 μm. **b** Melatonin-induced apoptosis of SGC-7901 gastric cancer cells. Values were presented as mean ± SD of three independent experiments. **c** Cell proliferation was measured by MTT assay. **d** Apoptosis was evaluated for up to 48 h by Hoechst 33258 staining. Images are representative of at least three independent determinations. *Scale bar* 50 μm. **P < 0.01

To assess the apoptosis effects of melatonin on gastric cancer cells, a widely used human gastric cancer cell line, SGC-7901, was employed. Clear evidence for apoptosis was obtained by monitored by fluorescence microscopy after staining with Hoechst 33258. As shown in Fig. 1a, b, in the control group, SGC-7901 cells exhibited regular shaped nuclei. In comparison, numbers of shrunken cells with condensed or fragmented nuclei, characteristic of apoptotic cells, were significantly increased in 1 mM melatonin treated cultures. We also treated cells with different doses of melatonin and cell viability was measured by MTT assay. As shown in Fig. 1c, the cell viability decreased gradually in a dose-dependent manner. We also assessed cell apoptosis in response to 1 mM melatonin treatment at 24 and 48 h, and SGC-7901 showed enhanced cell apoptosis in a time-dependent manner (Fig. 1d). These results showed that melatonin suppressed cell viability of SGC-7901 in vitro. The marked decrease of cell viability by 1 mM melatonin treatment most likely reflected the induction of cell apoptosis by melatonin. Thus, 1 mM melatonin was used for further apoptosis studies.

### Knockdown of HSP27 aggravates melatonin-induced cell apoptosis

**Fig. 2:**
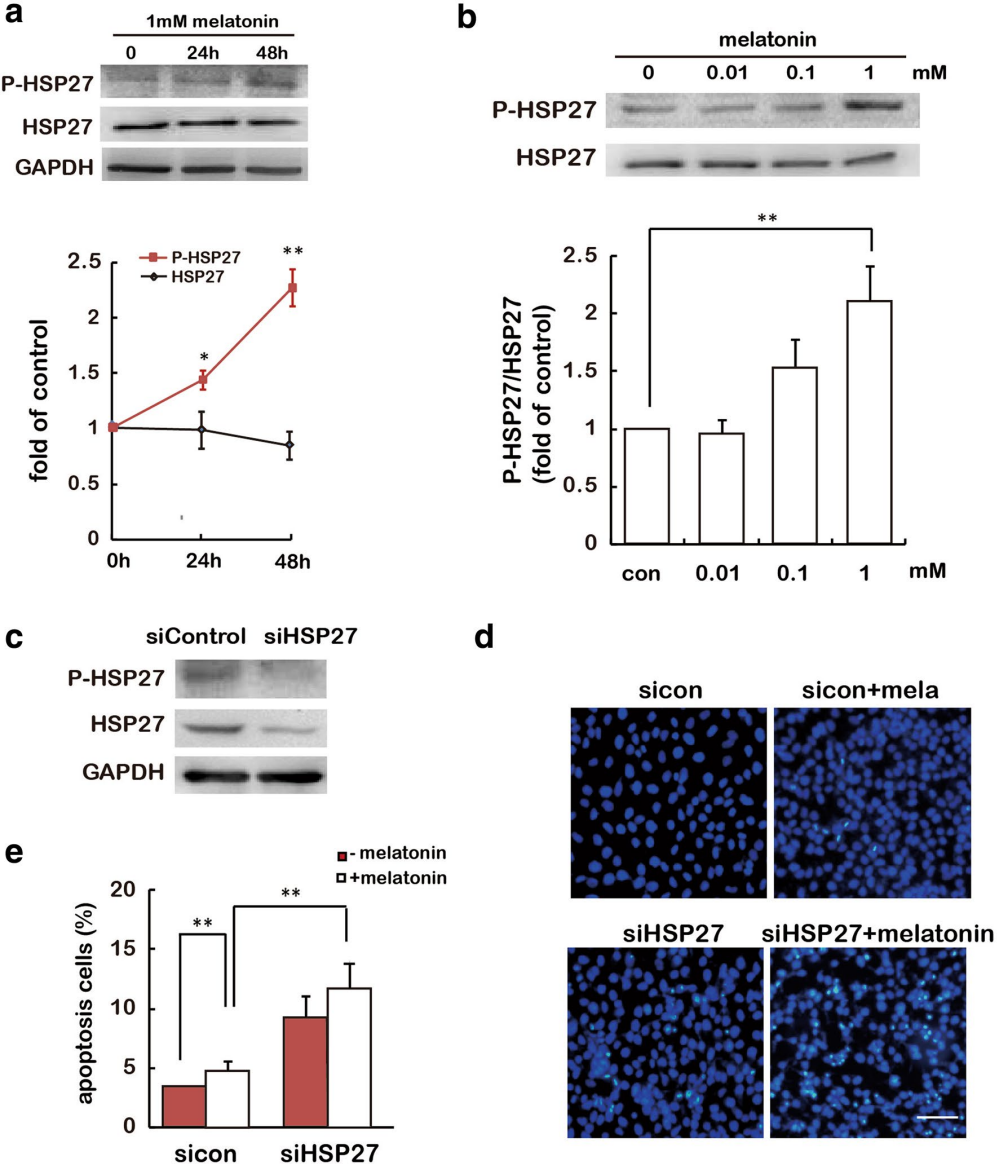
HSP27 phosphorylation is required for resistance of apoptosis by melatonin. **a**, **b** SGC-7901 cells were treated with melatonin for the indicated periods (**a**) and doses (**b**), and HSP27 phosphorylation was determined by immunoblotting assay. GAPDH was used as a loading control. *P < 0.05, **P < 0.01, referring to the difference between cells treated with and those without melatonin. **c** The expression of total and phosphorylation level of HSP27 after treatment with HSP27 siRNA. Total protein extracts from SGC-7901 cells transfected with HSP27 siRNA or control siRNA were analyzed by immunoblotting for P-HSP27 and HSP27. GAPDH was used as a loading control. **d**, **e** Cell apoptosis was determined by Hoechst 33258 staining in the SGC-7901 cells transfected with control siRNA or HSP27 siRNA. **d** Images are representative of at least three independent determinations. *Scale bar* 50 μm. **e** HSP27 siRNA treatment enhanced melatonin-induced apoptosis of SGC-7901 gastric cancer cells. Values were presented as mean ± SD of three independent experiments. **P < 0.01

To explore the mechanism whereby melatonin stimulates gastric cancer cell apoptosis, we first examined endogenous HSP27 activation after melatonin treatment. Melatonin treatment resulted in a time-dependent increase in HSP27 activity, as determined by immunoblotting analysis with an antibody against the phosphorylated form of HSP27. In contrast, the levels of total HSP27 were constant at all these time points (Fig. 2a). We also treated cells with different doses of melatonin (0.01–1 mM) for 24 h, and found that melatonin dose-dependently activated HSP27 in SGC-7901 cells (Fig. 2b). To determine the involvement of HSP27 in melatonin stimulated apoptosis of gastric cancer cells, specific siRNA for HSP27 were applied to SGC-7901 cells. As shown in Fig. 2c, HSP27 siRNA greatly knocked down total and phosphorylated HSP27 expression. Treatment of cells with 1 mM melatonin increased the apoptosis of SGC-7901 cells when compared with control cells. As expected, HSP27 knockdown not only resulted in a significant increase of melatonin-stimulated cell apoptosis, but also induced cell apoptosis in the absence of melatonin treatment in SGC-7901 cells (Fig. 2d, e). These results indicate that the knockdown of HSP27 may aggravate both basal and melatonin-stimulated gastric cancer cell apoptosis.

### PI3K/Akt mediates HSP27-induced resistance to apoptosis induced by melatonin

**Fig. 3:**
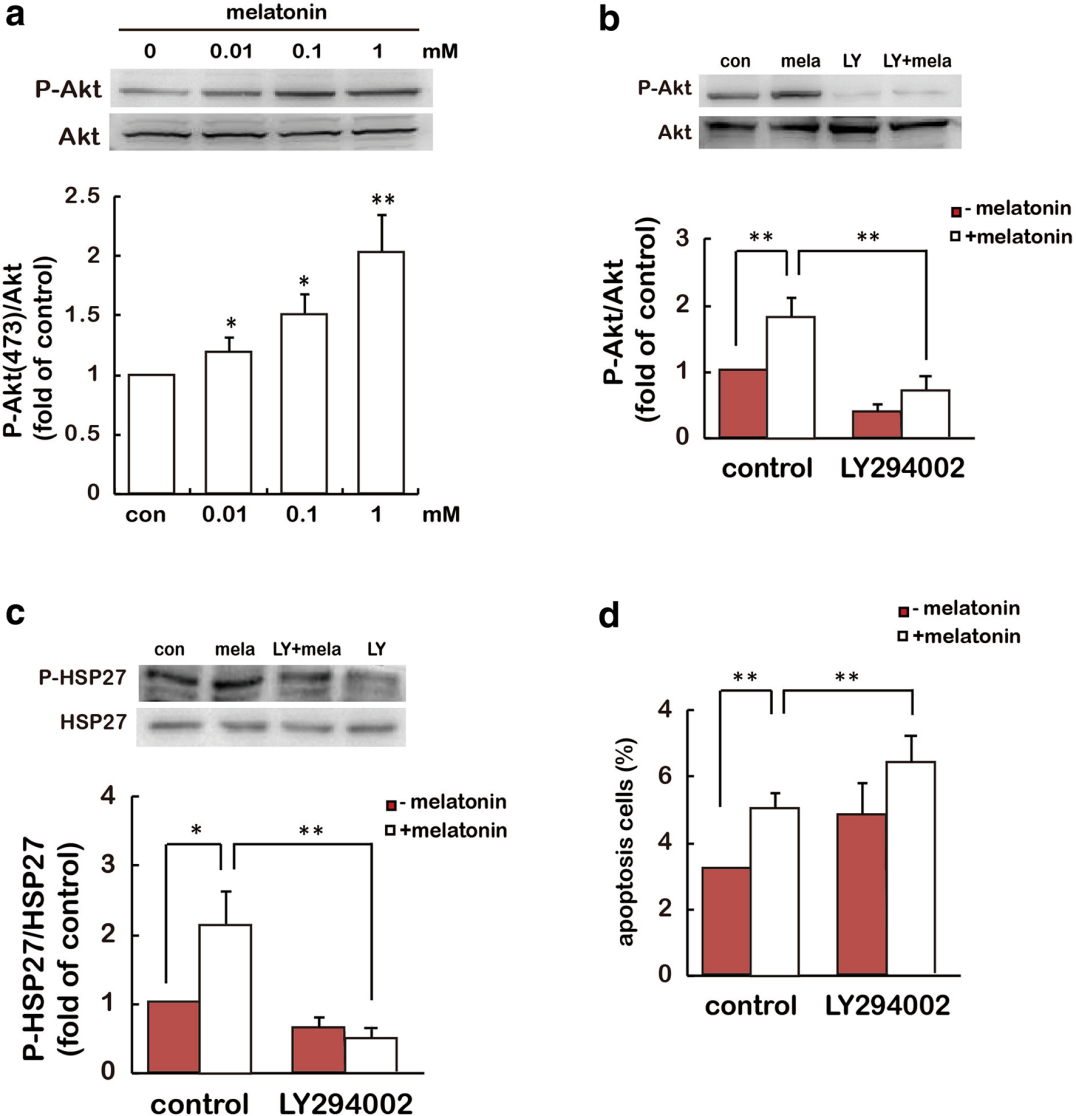
HSP27 enhanced cell resistance to melatonin-induced cell apoptosis is mediated through PI3K/Akt. **a**, **b** Effect of melatonin stimulation on phosphorylation of Akt. SGC-7901 cells were treated with melatonin under different concentrations of melatonin for 24 h. Melatonin-stimulated phosphorylation of Akt at Ser473 was determined by immunoblotting assay. GAPDH was used as a loading control. *P < 0.05, **P < 0.01, referring to the difference between cells treated with and those without melatonin. **b**, **c** After treatment with 10 μM PI3K inhibitor LY294002 for 30 min, SGC-7901 cells were stimulated with 1 mM melatonin for 24 h and then cells were analyzed by P-Akt (**b**) and P-HSP27 (**c**) expression, and **d** the effect of LY294002 on the melatonin-stimulated apoptosis in SGC-7901 cells was determined. *P < 0.05, **P < 0.01

To determine whether PI3K/Akt is the upstream mediator of HSP27 activation by melatonin in our system, immunoblotting analysis of P-Akt (Ser473), a well accepted downstream target of PI3K, was used to determine the PI3K activity [19, 20]. The results revealed a dose-dependent increase in PI3K activity following melatonin treatment. Immunoblotting analysis showed that the amount of phosphorylated Akt was increased significantly after melatonin stimulation with maximal activation at 1 mM (Fig. 3a). To determine whether melatonin-stimulated HSP27 activity is PI3K/Akt-dependent, we blocked PI3K/Akt activity by treating the cells with LY294002, a PI3K inhibitor (Fig. 3b), and examined HSP27 activity after stimulation with melatonin. The results showed that pretreatment with 10 μM LY294002 largely inhibited melatonin-induced Akt and HSP27 phosphorylation in comparison with control cells (Fig. 3c). The effect of PI3K inhibitor on cell apoptosis was also investigated using Hoechst 33258 assay. Pretreatment with 10 μM LY294002 resulted in a remarkable enhancer of both basal and melatonin-promoted cell apoptosis (Fig. 3d). These results suggest that PI3K/Akt acts upstream of HSP27 in resisting melatonin-stimulated gastric cancer cell apoptosis.

### P38 activation is required for melatonin-induced PI3K/Akt/HSP27 activation

**Fig. 4:**
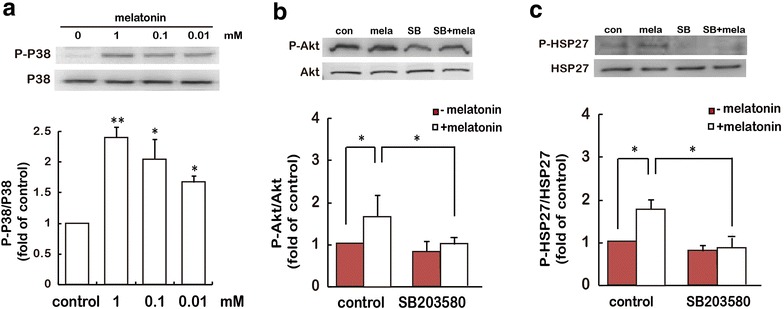
P38 is required for the activation of PI3K/Akt and HSP27 by melatonin. **a** SGC-7901 cells were treated with melatonin for the indicated doses for 24 h, and HSP27 phosphorylation was determined by immunoblotting assay. *P < 0.05, **P < 0.01, referring to the difference between cells treated with and those without melatonin. **b**, **c** After treatment with 20 μM P38 inhibitor SB203580 for 30 min, SGC-7901 cells were stimulated with 1 mM melatonin for 24 h, then the cells were analyzed by P-Akt (**b**) and P-HSP27 (**c**) expression. *P < 0.05

We also examined the effect of melatonin on P38 activation in cultured gastric cancer cells. Melatonin treatment resulted in a dose-dependent increase in P38 activity, as determined by immunoblotting with an antibody against the phosphorylated form of P38 (Fig. 4a). To investigate whether P38 activation leads to melatonin-induced activation of the PI3K/Akt/HSP27 signaling pathway and cell apoptosis in cultured gastric cancer cells, SB203580, a known inhibitor of P38, was used and its effect on melatonin-induced PI3K/Akt/HSP27 activation was examined. Pretreatment with 20 μM SB203580 remarkably abolished melatonin-induced PI3K/Akt (Fig. 4b) and HSP27 activation (Fig. 4c). These results indicate that melatonin can stimulate P38 activation in gastric cancer cells, and which may be an important mechanism for the stimulation of PI3K/Akt/HSP27 signaling pathway by melatonin.

**Fig. 5:**
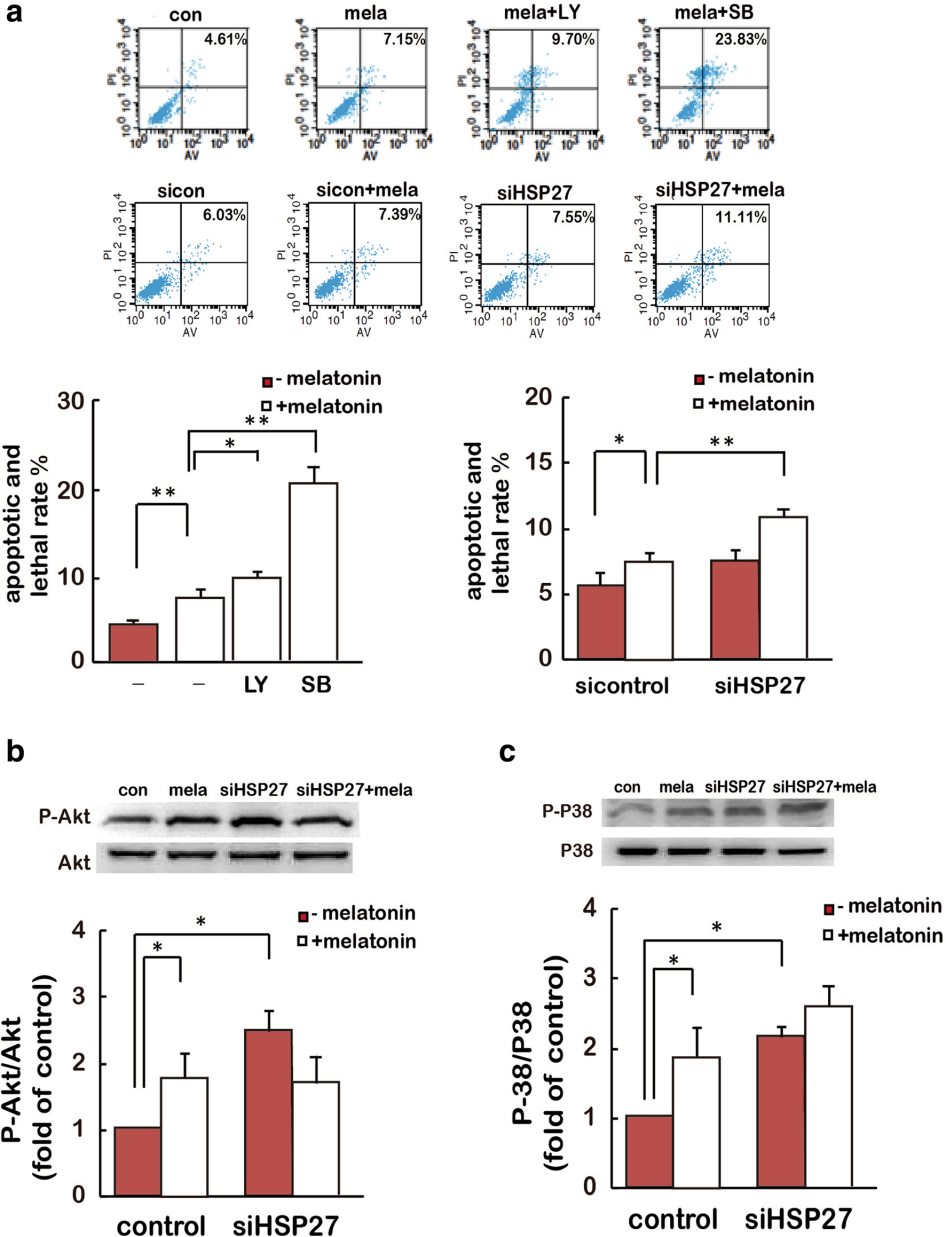
Effects of SB203580, LY294002 and HSP27 silencing on melatonin-induced cell apoptosis. **a** Effects of SB203580, LY294002 and siHSP27 on melatonin stimulated cell apoptosis and necrosis were measured with FACS assay. *P < 0.05, **P < 0.01. **b**, **c** After incubation with melatonin for 24 h, total protein extracts from SGC-7901 cells transfected with HSP27 siRNA or control siRNA were analyzed by immunoblotting for P-Akt (**b**) and P-P38 (**c**). *P < 0.05

Furthermore, by FACS analysis, we noticed that melatonin stimulated cell apoptosis was upregulated significantly by the pretreatment with LY294002 and SB203580, HSP27 knockdown also resulted in a significant increase of melatonin-stimulated cell apoptosis (Fig. 5a). In the meantime, we noted that HSP27 silencing not only has no effect on melatonin stimulated P-Akt (Fig. 5c) and P-P38 (Fig. 5d) level, but also induced P-P38 and P-Akt expression in the absence of melatonin treatment. These results indicate that the activation of HSP27 is not essential for both basal and melatonin-stimulated gastric cancer cell P38 and PI3K/Akt activation. Together, all the data above suggest a model that melatonin induces P38 activation, which stimulated activation of PI3K/Akt/HSP27 signaling for anti-apoptosis initiation (Fig. 5e).

## Discussion

Besides its role in preventing angiogenic tumor cells dormancy [19], HSP27 is also essential in maintaining normal homeostasis and morphology of malignant cells in response to stress conditions. Previous studies have shown that the susceptibility of gastric ulceration induced by NSAID was reduced in the transgenic mice which were overexpressed HSP27 [20]. Although HSP27 phosphorylation contributes to arachidonic acid-induced apoptosis in brain endothelial cells [21], here, we reported that, in SGC-7901 gastric cancer cells, melatonin stimulated HSP27 phosphorylation dose-dependently. Importantly, depletion of HSP27 resulted in an increase in the number of melatonin-induced apoptosis cells, indicating that HSP27 is actively involved in maintaining homeostasis of SGC-7901 cells by inhibiting cell apoptosis. This is consistent with the observation that melatonin stimulates accumulation of nuclear phosphoHSP27 in human pancreatic carcinoma cells [22]. The reasons for these divergent cellular outcomes are presently unclear, but HSP27 interaction with distinct substrates, or binding partners after different stimulator treatment, might be able to explain the opposing responses on cell apoptosis. Actually, HSP27 could regulate apoptosis through an ability to interact with multiple points of the apoptotic signaling pathway. For example, the phosphoHSP27 directly interacts with Daxx, preventing the interaction of Daxx with both Ask1 and Fas that mediates cell apoptosis [23]. Collectively, these findings attest to phosphoHSP27 being a potentially resistant molecule in apoptosis induced by melatonin.

We next examined the potential activators for HSP27 in our system. Akt is a Ser/Thr kinase and downstream mediator of the PI3K pathway regulating cell survival, differentiation, and growth factor responsiveness. The frequent aberrant activation of the PI3K/Akt pathway in human cancer has made it an attractive therapeutic target [24]. In granular keratinocytes, Akt activation not only induced HSP27 phosphorylation, but also changes the equilibrium between cytoplasmic and nuclear phosphorylated HSP27, leading to its greater concentration in the cytoplasm [25]. In fact, it is reported that activation of Akt induced by phosphorylated HSP27 confers the apoptosis–resistance in t-AUCB-treated glioblastoma cells in vitro [26]. Our results show that melatonin induces a dose-dependent increase in PI3K/Akt activity. Blocking PI3K activity by LY294002 significantly prevents melatonin-induced HSP27 activation and aggravates cell apoptosis. Therefore, it may be reasonable to think that activation of PI3K/Akt signaling upon treatment with melatonin increased HSP27 phosphorylation, which was resistant to melatonin chemotherapy.

Several reports have demonstrated that HSP27 can form multicomponent complexes with Akt, and P38, and this complex has been shown to be involved in controlling HSP27 phosphorylation and stress-induced apoptosis [17, 27, 28]. As reported previously, activation of P38 was accompanied by Akt suppression in the apoptotic process in some types of cancer cells [29, 30]. Conversely, there also has a good evidence suggesting a link from MEKK4 and P38, via HSP27, to Akt activation, which is essential for the regulation of cell fate in response to apoptotic stress in human prostate cancer cells [31]. However, in gastric cancer cells, whether P38 might affect the PI3K/Akt and HSP27 activation induced by melatonin is unknown. Here, our results clearly reveal that melatonin triggers a rapid stimulation of P38 activity. The selective P38 inhibitor SB203580 effectively blocks melatonin-stimulated PI3K/Akt and HSP27 activation. Consequently, we conclude that P38 is an upstream component of PI3K/Akt/HSP27 signaling pathway in melatonin-stimulated apoptosis in SGC-7901 gastric cancer cells.

Then another important question arises about the relationship between the activation of P38 and anti-apoptosis of PI3K/Akt/HSP27 after melatonin treatment. Accumulating evidence suggests that P38 could be strongly activated by environmental and genotoxic stresses [32]. Notably, P38 plays a dual role in stress-induced apoptosis. On the one hand, many chemotherapeutic agents including melatonin require P38 activity for the induction of apoptosis [33], on the other hand, the P38/HSP27 axis plays an important role in mediated drug resistance in cancer treatment [34]. Accordingly, we found that when P38 activity was blocked by its inhibitor, melatonin-stimulated cell apoptosis was dramatically increased, indicating that P38 activation is resistant to melatonin-induced apoptotic process. Adding to the results that P38 inhibitor could also suppress PI3K/Akt and HSP27 activation, these findings suggest that P38, through its regulation of PI3K/Akt/HSP27 anti-apoptosis pathway, serves as a suppressor of melatonin-stimulated SGC-7901 cells apoptosis.

Intriguingly, in contrast to the well-established pro-apoptotic roles of melatonin, our study revealed clearly a distinguishable anti-apoptotic pathway induced by melatonin. Taken together, all these results demonstrate that HSP27 plays a crucial role in apoptotic resistance in melatonin-treated SGC-7901 gastric cancer cells, and its activation is most likely via the activation of P38/PI3K/Akt signaling by melatonin. These findings are of potential pathophysiological importance for understanding the integration of melatonin-related signaling and further substantiate the molecular basis for clinical trials applying melatonin for the treatment of gastric cancer.
